# Cyborg beast: a low-cost 3d-printed prosthetic hand for children with upper-limb differences

**DOI:** 10.1186/s13104-015-0971-9

**Published:** 2015-01-20

**Authors:** Jorge Zuniga, Dimitrios Katsavelis, Jean Peck, John Stollberg, Marc Petrykowski, Adam Carson, Cristina Fernandez

**Affiliations:** Department of Exercise Science and Pre Health Professions, Creighton University, Omaha, NE 68178 USA; CHI Health Creighton University Medical Center, Omaha, NE 68131 USA; Department of Occupational Therapy, Creighton University, Omaha, NE 68178 USA; Children’s Hospital and Medical Center, Omaha, NE 68114 USA

**Keywords:** 3D printing, Computer-aided design, Low-cost prosthesis, Custom-made prosthesis, Prosthesis for children

## Abstract

**Background:**

There is an increasing number of children with traumatic and congenital hand amputations or reductions. Children's prosthetic needs are complex due to their small size, constant growth, and psychosocial development. Families’ financial resources play a crucial role in the prescription of prostheses for their children, especially when private insurance and public funding are insufficient. Electric-powered (i.e., myoelectric) and body-powered (i.e., mechanical) devices have been developed to accommodate children’s needs, but the cost of maintenance and replacement represents an obstacle for many families. Due to the complexity and high cost of these prosthetic hands, they are not accessible to children from low-income, uninsured families or to children from developing countries. Advancements in computer-aided design (CAD) programs, additive manufacturing, and image editing software offer the possibility of designing, printing, and fitting prosthetic hands devices at a distance and at very low cost. The purpose of this preliminary investigation was to describe a low-cost three-dimensional (3D)-printed prosthetic hand for children with upper-limb reductions and to propose a prosthesis fitting methodology that can be performed at a distance.

**Results:**

No significant mean differences were found between the anthropometric and range of motion measurements taken directly from the upper limbs of subjects versus those extracted from photographs. The Bland and Altman plots show no major bias and narrow limits of agreements for lengths and widths and small bias and wider limits of agreements for the range of motion measurements. The main finding of the survey was that our prosthetic device may have a significant potential to positively impact quality of life and daily usage, and can be incorporated in several activities at home and in school.

**Conclusions:**

This investigation describes a low-cost 3D-printed prosthetic hand for children and proposes a distance fitting procedure. The Cyborg Beast prosthetic hand and the proposed distance-fitting procedures may represent a possible low-cost alternative for children in developing countries and those who have limited access to health care providers. Further studies should examine the functionality, validity, durability, benefits, and rejection rate of this type of low-cost 3D-printed prosthetic device.

## Background

Children’s prosthetic needs are complex due to their small size, constant growth, and psychosocial development [1]. Familial financial resources play a crucial role in prescription of prostheses for children, especially when private insurance and public funding are insufficient [1]. Most upper-limb prostheses include a terminal device, with the objective to replace the missing hand or fingers. The cost of a body-powered prosthetic hand ranges from $4,000 to $20,000; depending on the mode of control, these devices require extensive fitting procedures to develop the terminal device and often include a complex system of cables and harnesses [2]. Electric-powered units (i.e., myoelectric) and mechanical devices (i.e., body-powered) have been improved to accommodate children’s needs, but the cost of maintenance and replacement represents an obstacle for many families. Voluntary-closing upper-limb prosthetic devices are more suitable for children [1,3] and play a crucial role in improving gross motor development [1]. Currently, the most cost-effective option for pediatric populations is a passive prosthetic hook [1]; although functional, these devices have a high rejection rate, in part due to an unacceptable cosmetic appearance [4-6]. Most current prosthetics do not adapt to the normal growth of children’s limbs and require constant visits to health care providers for adjustments or replacement, which may lead to abandonment [1,6].

There has been an increase in the number of children born with congenital upper-limb deficiencies or acquired traumatic amputations during the past two decades [7-9]. It is estimated that, in the United States, more than 32,500 children suffer from a major pediatric amputation [8], and the Centers for Disease Control and Prevention estimates that about 1,500 children are born with upper-limb reductions in the United States each year [9]. Worldwide estimates for upper-limb reductions range from 4-5/10,000 to 1/100 live births [7]. There is a critical need for practical, easy-to-replace, customized, aesthetically appealing, low-cost prosthetic devices for children [10].

**Figure 1:**
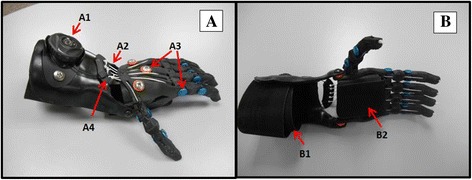
**Prosthetic hand (Cyborg Beast). A**: Top view (A1: Tensioner dial, A2: Lift nylon cords, A3: Chicago screws, A4: Tension balance system) and **B**: Bottom view (B1: Forearm adjustable Velcro strap, B2: Hand adjustable Velcro strap).

Advancements in computer-aided design (CAD) programs, additive manufacturing, and open source image editing software offer the possibility of designing, printing, and fitting prosthetic hands and other assistive devices at very low cost [11] (Figure 1). The development of low-cost prosthetic devices with practical and easy fitting procedures that can be performed at a distance would have a significant clinical and social impact on children around the world.

### Research Purpose

The aim of this preliminary investigation was to briefly describe a low-cost three-dimensional (3D)-printed prosthetic hand for children with upper-limb reductions and to propose a prosthesis fitting methodology that can be performed at a distance. We hypothesized that anthropometric measurement of the upper limbs taken from photographs and processed by image editing software would not differ from anthropometric measurements taken directly on upper limbs.

## Methods

### Subjects

Eleven children (two girls and seven boys, 3 to 16 years of age) with upper-limb reductions (one traumatic and eight congenital) participated in this study and were fitted with a low-cost 3D-printed prosthetic hand. Of the 11 participants, nine performed the laboratory visits and two were distance participants. A comparison between anthropometric measurements of the upper limbs taken from photographs and those taken directly on the upper limbs were reported for only nine local participants. Inclusion criteria included boys and girls from 3 to 17 years of age with unilateral carpus upper-limb reductions, missing some or all fingers, and wrist range of motion of the affected wrist greater than 20°. Exclusion criteria included upper extremity injury within the past month and any medical conditions that would contraindicate the use of our prosthetic hand prototype, such as skin abrasions and musculoskeletal injuries. The study was approved by the Creighton University Institutional Review Board and all the subjects completed a medical history questionnaire. All parents and children were informed about the study and parents signed a parental permission form. For children 6 to 16, an assent was explained by the principal investigator and signed by the children and their parents. Written informed consent from the parents was also obtained in order to publish the images shown in the present investigation. In addition, detailed safety guidelines were given to the parents regarding the use and care of the prosthetic hand.

### 3D-printed prosthetic hand characteristics and usage

The low-cost 3D-printed prosthetic hand named “Cyborg Beast” (Figure 1) was designed using a modeling software program (Blender 7.2, Blender Foundation, Amsterdam, Netherlands) and manufactured in the researcher’s laboratory using desktop 3D printers (Makerbot Replicator 2X, Makerbot Industries, Brooklyn, NY, and Ultimaker 2, Ultimaker B.V., Geldermalsen, The Netherlands). Elastic cords placed inside the dorsal aspect of the fingers provide passive finger extension. Finger flexion is driven by non-elastic cords along the palmar surface of each finger and is activated through 20-30° of wrist flexion. The result is a composite fist (flexing the fingers towards the palm) for gross grasp. The materials used for printing our prosthetic hand are polylactide (PLA) plastic and acrylonitrile butadiene styrene (ABS). Other components of the prosthetic hand include Chicago screws of various sizes, 1 mm lift nylon cord, 1.5 mm elastic cord, Velcro, medical-grade firm padded foam, protective skin sock, and a dial tensioner system (Mid power reel M3, Boa Technology Inc., Denver, Colorado). The majority of these materials are available at local hardware stores or online. The present cost of materials is about $50 USD. The average time to fully assemble the prosthetic hand design is approximately 2. 5 hours. The weight of a fully assembled hand at a 140% of its original size is 184.2 grams. A similar device costs approximately $4,000 and weighs about 400 grams. The Cyborg Beast prosthetic hand is well suited for activities that involved the manipulation of light objects using lateral, power (composite), and spherical prehensile patterns.

**Figure 2:**
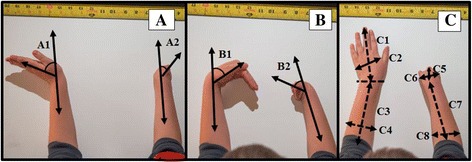
**Three photographs of upper limbs. A**: wrist extension (A1: non-affected, A2 affected), **B**: wrist flexion (B1: non-affected, B2: affected), and **C**: Top view (C1: Non-affected hand length, C2: Non-affected hand width, C3: Non-affected forearm length, C4: Non-affected forearm width, C5: Affected hand length, C6: Affected hand width, C7: Affected forearm length, and C8: Affected forearm width).

Justification for the design and use of the 3D-printed prosthetic hand are low cost, easy usage, easy fitting, easy assembly, and visually appealing to children. The fitting procedures for the prosthetic hand require a few simple anthropometric measures of both limbs (Figure 2) to properly scale the prosthetic device. The files for the design are available online on the National Institutes of Health (NIH) 3D print exchange website (http://3dprint.nih.gov/discover/3dpx-000524) and Thingiverse (http://www.thingiverse.com/thing:261462). All families and children participating in this study completed a short survey. The survey was developed to estimate the impact of this prosthetic device, including items related to quality of life, daily usage, and types of activities performed. The survey has not been statistically validated, but provides useful information related to usage and perception about improvements in quality of life. After approximately one to three months of using the prosthetic hand, 11 children and their parents reported some increases in quality of life (four indicated this was significant and seven indicated a small increase), while one indicated no change. Nine children reported using the hand one to two hours a day, three reported using the prosthetic hand more than two hours, and one reported using the hand only when needed. Furthermore, children reported using the prosthetic hand just for fun (n = 10), for activities at home (n = 9), to play (n = 6), for school activities (n = 4), and to perform sports (n = 2).

### Proposed distance-fitting procedure

The prosthetic hand (Figure 1) was designed to allow easy fitting with minimal anthropometric measurement requirements, which include hand length (tip of the middle finger to center of the wrist joint, Figure 2C1and C5), palm width (widest region of the palm above the base of the thumb, Figure 2C2), forearm length (center of the wrist joint to center of the elbow joint, Figure 2C3 and C6), forearm width at three-fourths (width of the forearm at proximal three-fourths of the length of the forearm proximal to the wrist, Figure 2C4 and C7), and range of motion of the wrists (extension and flexion, Figure 2A1 and A1). The proposed distance-fitting procedure involves extracting all these required measurements from three photographs of the upper limbs (Figure 2).

To compare the anthropometric measurements taken directly from the subject’s upper limbs with those extracted from photographs, a trained occupational therapist took the required anthropometric measurements directly from the subject’s upper limbs using a standard tape measure and goniometer. Three photographs of the upper limbs were taken as shown in Figure 2. All pictures included a ruler and were taken directly above the arms and included the entire forearm up to the elbow. To measure range of motion of the wrist, participants extended (Figure 2A) and flexed (Figure 2B) their wrists as far as possible. In addition, a reference line was drawn over the participant’s wrist joint of the non-affected hand (Figure 2C). An image editing program (ImageJ, version 1.46, NIH) was used to assess hand length, palm width, forearm length, forearm width at three-fourths, and range of motion of the wrists for flexion and extension (Figure 2). All anthropometric measurements were taken directly from the subject’s upper limbs and compared to those extracted from photographs using an image editing program. All measurements were expressed in centimeters and calibrated using the ruler included in the image.

**Figure 3:**
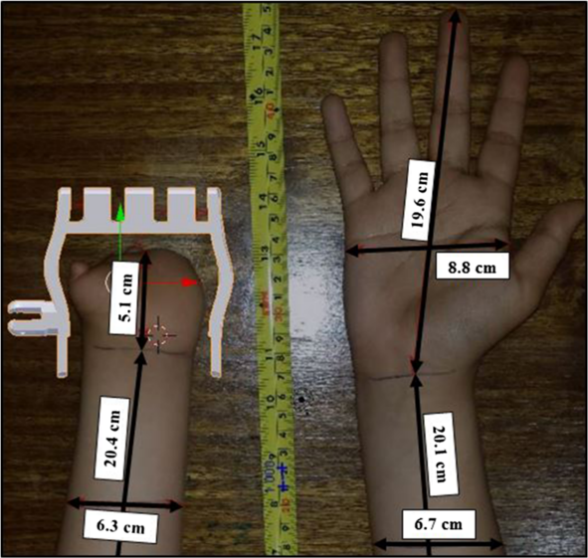
**Illustration of an image imported as plane and a Cyborg beast palm scale at 140% for a 16-year-old research participant.**

**Figure 4:**
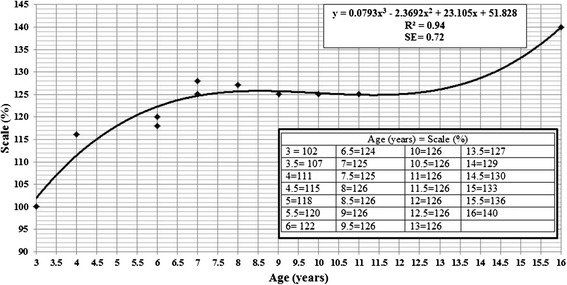
**Sizing chart for Cyborg Beast prosthetic hand. Instructions: locate the child’s age in the bottom (X axis) and follow the line to the regression curve and then locate the intercepting line corresponding to the scale % on the left side (Y axis).** Example: For a 5-year-old, the scale % of the Cyborg Beast would be 118% (±1.44%). This cubic regression equation was derived from a mixed sample of 11 children with ages ranging from 3 to 16 years of age.

After saving the images files with the calibrated measurements, they were imported as planes in Blender (Figure 3). Calibration of the metric scale on Blender was performed by changing the default unit (meter) to centimeters by adjusting the scale to 0.001. The image plane was resized to match the size of the 1 cm background grid on Blender using the ruler on the imported image plane. The accuracy of the calibrations was confirmed using the interactive ruler tool on Blender, performing several measurements over the ruler included in the image plane (Figure 3). After the image plane was calibrated, a sizing chart was used to estimate the predicted size of the prosthetic hand expressed as a percentage of its original size (Figure 4). MakerWare software (Makerbot Industries, Brooklyn, NY) was used to size the prosthetic hand to the desired scale (%) using the scaling function. The sizing chart was developed to provide an easy method to scale the prosthetic hand for the user with no previous knowledge of CAD programs. For cases in which the cubic regression equation (Figure 4) was not able to accurately predict the correct size of the prosthetic hand due to differences in hand morphology, customized adjustments were made on Blender to ensure the proper fit. All the fitting procedures were performed with the assistance of an occupational hand therapist and a prosthetist. Thus, it is recommended to include clinical experts in the process of fitting the prosthetic device to avoid skin abrasions or breakdown due to improper fit.

### Statistical Analysis

#### Anthropometric Measurements

Seven separate two-way repeated measures ANOVAs [2 × 2; hand (affected versus non-affected) × fitting procedures (direct versus photographs)] were performed to analyze the data. In addition, the data have also been presented using the method of Bland and Altman as described by previous investigations [12-14]. Pearson product–moment correlation coefficient was calculated to examine the correlations between the difference and the mean of the difference from the mean values shown in the Bland and Altman plots. A p-value of ≤0.05 was considered statistically significant for all comparisons.

## Results

**Figure 5:**
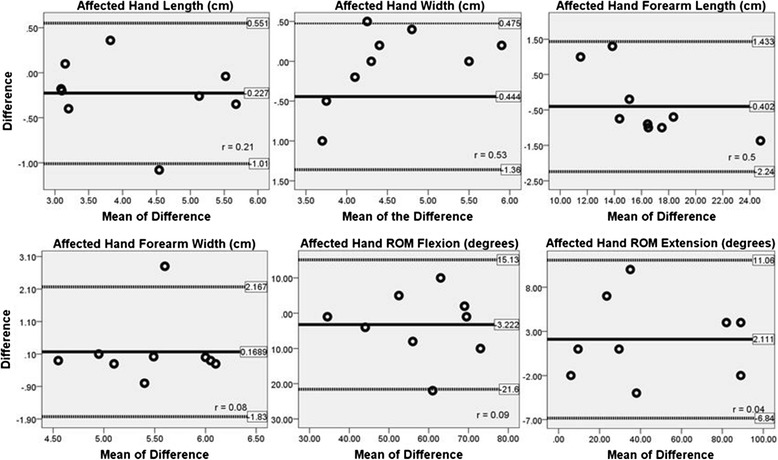
**Bland and Altman plots for anthropometric and range of motion measurements taken directly from the subject’s upper limbs and those taken from photographs.**

The results of the two-way repeated measures ANOVAs showed no significant mean difference between the anthropometric measures taken directly on the subject’s upper limbs and those taken from the photographs (Table 1). There were no significant two-way interactions for repeated measures ANOVAs performed for hand × fitting procedures. There was a significant main effect, however, for hand (affected versus non-affected), with no significant main effect for fitting procedures (direct versus photographs). When the relationship between scale of the prosthetic hand (%) versus age (years) was analyzed, our results indicated that the cubic model was the best-fit for our sample (Figure 4). The main finding of the survey was that our prosthetic device may have a significant potential to positively impact quality of life and daily usage, and can be incorporated in several activities at home and in school. The Bland and Altman plots (Figure 5) show 95% limits of agreements for the anthropometric measurements of the affected hand and measures of range of motion. The average discrepancy (represented by a solid line in Figure 5) for the lengths and widths of the hand and forearm resulted in values close to zero, indicating no major bias. The limits of agreement (represented by a dotted line in Figure 5) are narrow and show that these measures tend to be within 5 mm of each other. The range of motion measurements, however, presented a small bias (average discrepancy values greater than zero) and wider limits of agreements, with about 10° difference between methods. No trends were found and the correlations between the difference and mean of the difference were not significant, ranging from 0.04 to 0.53 (Figure 5).

**Table 1:** **Mean (±SD) for anthropometric measures and range of motion of the wrists**

**Measurements**	**Non-affected**		**Affected**	
	**Direct**	**Photographs**	**Direct**	**Photographs**
Hand Length (cm)	13.83 ± 2.44	13.44 ± 1.73	4.02 ± 1.07	4.25 ± 1.15
Palm Width (cm)	7.00 ± 1.20	6.91 ± 0.95	4.50 ± 0.90	4.54 ± 0.66
Forearm Length (cm)	18.94 ± 3.88	18.94 ± 4.16	16.29 ± 3.41	16.69 ± 4.09
Forearm Width (cm)	6.23 ± 0.85	6.47 ± 1.12	5.57 ± 0.77	5.54 ± 0.59
Wrist Range of Motion Flexion (°)	76.00 ± 10.27	75.33 ± 11.01	56.44 ± 13.15	59.76 ± 13.95
Wrist Range of Motion Extension (°)	76.44 ± 5.7	76.00 ± 6.96	45.67 ± 33.47	43.56 ± 33.29

The results of the two-way repeated measures ANOVAs showed no significant (p >0.05) mean difference between the anthropometric measures taken directly on the subject’s upper limbs and those taken from photographs. There were no significant two-way interactions for repeated measures ANOVAs performed for hand x fitting procedures. There was a significant main effect for hand (affected versus non-affected), with no significant main effect for fitting procedures (direct versus photographs).

## Discussion

The results of the present investigation indicated that there were no mean differences between anthropometric measures taken directly from the subject’s upper limbs and those extracted from photographs (Table 1). The Bland and Altman plots (Figure 5) show no major bias and narrow limits of agreements for lengths and widths and small bias and wider limits of agreements for the range of motion measurements. Furthermore, the survey indicated that the prosthetic device may have a significant potential to positively impact quality of life and daily usage in several activities at home or school. The fitting procedures of our prosthetic hand design require minimal anthropometric measurements of the upper limbs for proper scaling and fitting. Most fitting procedures required for prosthetic hands include wrap casting using plaster bandages placed over the affected limb [2]. More recently, 3D scanning has also been used for the development of different type of prostheses and orthoses [11,15,16]. Casting procedures require the physical presence of the individual needing the prosthetic hand and the health care professional in the same physical location, which may not be possible for patients living in rural or isolated areas. 3D scanning procedures required sophisticated equipment and technical knowledge to perform the measurements. Furthermore, both techniques require the patient to visit the health care facilities for proper fitting procedures.

The results from the present investigation provide a novel distance-fitting procedure for a low-cost 3D-printed prosthetic hand for children with upper-limb differences. Image editing software to extract information from digital images has been used for a wide range of disciplines, including molecular biology and archeology [17,18]. The present investigation applied image editing techniques to extract anthropometric data and 3D modeling applications to develop a novel distance-fitting procedure. The recent popularity and low cost of desktop 3D printers makes the prosthetic hand described in the current investigation readily accessible. The proposed distance-fitting procedures can make this device accessible to a great number of children in need of this type of device around the globe. These procedures, however, must be performed with caution, since inaccurate scaling or significant errors in the measurements could affect the function or fitting of the 3D-printed prosthetic hand. Overall, this low-cost prosthetic hand and the ability to fit this device at a distance represent a low-cost alternative for children in developing countries and children from uninsured or economically disadvantaged families.

## Conclusion

This investigation provides a description of a low-cost 3D-printed prosthetic hand for children and proposes a distance-fitting procedure. The Cyborg Beast prosthetic hand and the proposed distance-fitting procedure represent a possible low-cost alternative for children in developing countries and those with little or no access to health care providers. Our prosthetic device may have a significant potential to positively impact quality of life and daily usage. Further studies should examine the functionality, validity, durability, benefits, and rejection rate of this low-cost 3D-printed hand design.

### Consent

All parents and children were informed about the study and signed a parental permission. For children 6 to 16, an assent was explained by the principal investigator and signed by the children and their parents. Written informed consent from the parents was obtained in order to publish the images shown in the present investigation. Furthermore, detailed safety guidelines were given to the parents regarding the use and care of the prosthetic hand.

## Abbreviations

3D: Three-dimensional
CAD: Computer-aided design
ABS: Acrylonitrile butadiene styrene
PLA: Polylactide
ANOVA: Analysis of variance
